# Health Conditions, Lifestyle Factors and Depression in Adults in Qingdao, China: A Cross-Sectional Study

**DOI:** 10.3389/fpsyt.2021.508810

**Published:** 2021-05-14

**Authors:** Nan Cui, Jing Cui, Xinpeng Xu, Bilal Aslam, Lan Bai, Decheng Li, Di Wu, Zhongren Ma, Jianping Sun, Zulqarnain Baloch

**Affiliations:** ^1^The Affiliated Hospital of Qingdao University, Qingdao, China; ^2^Qingdao Municipal Center for Disease Control and Prevention, Qingdao Institute of Preventive Medicine, Qingdao, China; ^3^Research Center for Health Policy and Management, Nanjing University, Nanjing, China; ^4^Biomedical Research Center, Northwest Minzu University, Lanzhou, China; ^5^Department of Microbiology, Government College University, Faisalabad, Pakistan

**Keywords:** health conditions, lifestyle factors, depression, cross-sectional study, diabetes

## Abstract

**Background:** Depression is a common mental illness. Previous studies suggested that health conditions and lifestyle factors were associated with depression. However, only few studies have explored the risk factors of depression in a large representative sample of the general population in the world.

**Methods:** A population-based cross-sectional survey was conducted in the 2006 survey and 2009 survey in Qingdao, China. The participants with insufficient information were excluded: Zung score, body mass index (BMI), diabetes items, physical activity, smoking, or drinking. Finally, a total of 3,300 participants were included in this analysis. The category of depression was used in the Zung self-rating depression scale (ZSDS). The associations between different indicators of health conditions (diabetic status, BMI), lifestyle factors (physical activity, smoking, and alcohol consumption), and depression were assessed by the logistic regression model.

**Results:** The mean Zung scores for all participants, male participants, and female participants were 29.73 ± 7.57, 28.89 ± 7.30, 30.30 ± 7.70, respectively. In all participants, those who were pre-diabetes status (OR: 1.53, 95% CI: 1.04–2.27), and irregular physical activity (OR: 0.39, 95% CI: 0.17–0.89) had an increased risk of depression. In man, the analysis showed an increased risk of depression those with pre-diabetes (OR: 2.49, 95% CI: 1.25–4.97), previously diagnosed diabetes (OR: 4.44, 95% CI: 1.58, 12.48), and in those irregular activities (OR: 0.07, 95% CI: 0.01–0.61). In women, those who were underweight (OR: 5.66, 95% CI: 1.04–30.71) had a greater risk of depression.

**Conclusions:** These results suggested that health conditions and lifestyle factors were the potential risk factors for depression. Men with pre-diabetes, previously diagnosed diabetes, and irregular activity had an increased risk for depression; women with underweight status had a higher risk for depression.

## Introduction

Depression is a common mental illness that has a substantial impact on one's private and public life. Significant efforts have been made to embed the depression into the policy agenda as a top priority across the world. However, depression is still a major public health issue with continuously increasing prevalence rates; the World Health Organization predicted that depression would be the three leading contributors to the global burden of disease by 2030 (1). Depression is also a dramatic problem in China: in 2013, the prevalence of depression was ~25% and projected that the burden of depression would increase by 10% between 2013 and 2025 (2). Therefore, preventing the incidence of depression is extremely important in the field of public health.

To help prevent depression, several studies on the risk factors for depression have been identified, including health conditions (BMI, chronic status) (3–6), lifestyle factors (smoking, drinking, physical activity, diet) (7, 8), individual determinants and socio-demographics (age, gender, education, marital status) (9, 10). Also, gender differences have observed in risk factors: having a chronic disease and physical inactivity were risk factors for depression in men, poor perceived health and a BMI of 25 or more were risk factors for depression in women (11). However, few of these studies have explored the risk factors of depression in a large representative sample of the general population.

The association between health conditions, lifestyle factors and depression has been rarely studied in China; yet there is a need to explore the nature of the association between the risk factors and the development of depression. Considering the results of previous studies, we chose two health conditions indicators of diabetic status and BMI and three lifestyle indicators including physical activity, smoking, and alcohol consumption. In the present study, we used the large population-based cross-sectional survey to assess the relationship between health conditions, lifestyle factors, and depression among the general population of Qingdao, China.

## Methods

### Study Population

**Figure 1:**
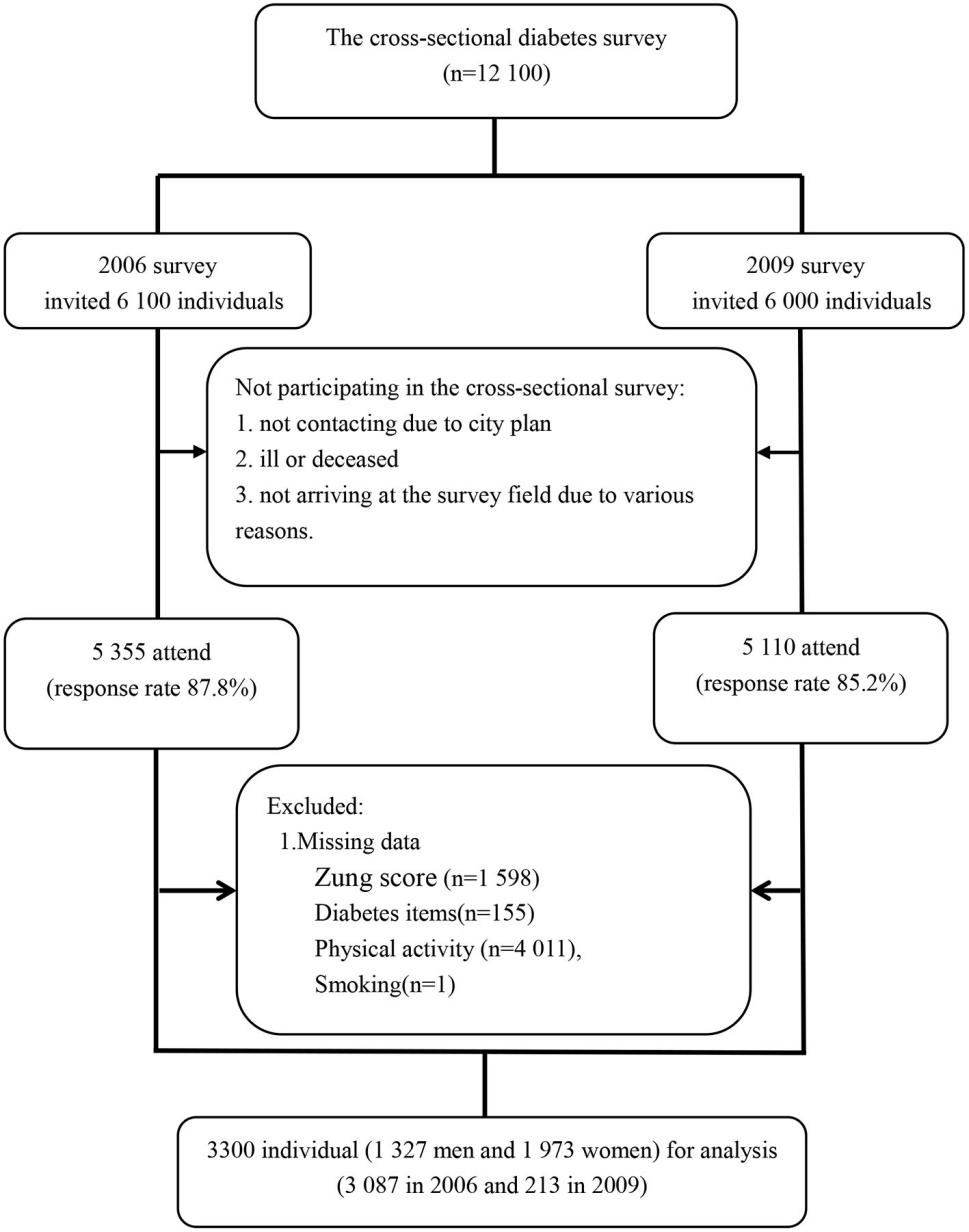
Flow chart of participants included in the cross-sectional analysis.

The exploited data from the Qingdao Diabetes Prevention Program (QDPP) were conducted in 2006 survey and 2009 survey in Qingdao, China. The diabetes survey covered three urban regions (Shinan, Shibei, Sifang,) and three rural regions (Huangdao, Jiaonan, and Jimo) in Qingdao. A total of 12,100 individuals (6,100 in 2006 survey and 6,000 in 2009 survey) were enrolled using the four-stage stratified random sampling method to select the representative sample aged 35–74 years old living in the selected area for more than 5 years. In the cross-sectional survey, 1,635 individuals (745 individuals in 2006 and 890 individuals in 2009) did not arrive at the survey field with or without various reasons, such as not contacting, ill or deceased. Finally, about 10,465 subjects (5,355 in 2006 survey and 5,110 in 2009 survey) were investigated in the survey, giving the response rate was 85.8% (87.8% in 2006 and 85.2% in 2009) (Figure 1).

From the 10,465 individuals, we excluded participants due to an insufficient information: 1,598 individuals without Zung score, 155 individuals without diabetes information, 4,011 individuals without physical activity (PA) information, 1 individual without smoking information. Finally, a total of 3,300 participants met the inclusion and exclusion criteria for this analysis.

### Depression

The dependent variable was the presence of depression symptoms, assessed using the Zung self-rating depression scale (ZSDS) (12). Compared to other tools, the ZSDS is more standardized for the self-administered scales to identify the characteristics of the depressive symptoms; therefore, it becomes one of the most frequently used self-administered scales (13). This scale contains 20 items rated on a scale of 1 through 4 based on the following responses: never or a little time, sometimes, most of the time, or almost always, and the total score ranges from 20 to 80; higher scores represent a higher level of depressive symptoms (14). The participants were categorized into two groups: normal (scores from 20 to 44), and depressed (scores ≥ 45).

### Health Conditions

Two items represented heath conditions: diabetic status and BMI. According to the 2006 World Health Organization/IDF standards (15), diabetic status was classified as normoglycaemia: a fasting plasma glucose (FPG) ≤6 mmol/L and 2 h post-load plasma glucose (2 h PG) ≤7.8 mmol/L; pre-diabetes: 6 mmol < FPG < 7 mmol/L or 7.8 mmol/L ≤ 2 h PG <11.1 mmol/L; newly diagnosed diabetes: FPG ≥ 7 mmol/L or 2 h PG ≥ 11.1 mmol/L, and previously diagnosed diabetes has been diagnosed as diabetes by an above the county level hospital before survey.

Body mass index (BMI) is calculated by dividing weight in kilograms by height in meters squared. According to WHO criteria participants were divided into three categorical groups: underweight (<18.5 kg/m^2^), normal weight (18.5–25 kg/m^2^), overweight/obese (>25 kg/m^2^).

### Lifestyle Factors

Three items assessed lifestyle factors: physical activity, smoking, and alcohol consumption. Physical activity was assessed by asking, “Are you doing regular physical activity now?” with possible answer of “yes” or “no.” Smoking status was assessed by asking, “Do you smoke?” with possible answer of “yes” or “no.” Drinking status was assessed by asking, “Do you drink?” with possible answer of “yes” or “no.”

### Adjusted Factors

The adjusted socio-economic and demographic variables showed as follows: gender, age, area, education, marriage, monthly income, occupation. The area was classified as urban and rural. Education was defined as illiterate/elementary school, junior high school, and senior high school or higher. Marriage was developed two dichotomous categories: unmarried (single, widowed, divorced, or separated) vs. married/cohabiting. Personal monthly income was coded as three levels factor: ≤599 Chinese Yuan (CNY), 600–1,999 CNY, ≥2,000 CNY. The occupation was classified into any kind of occupation (salaried employee, retired, self-employed) and no occupation (unemployed, never worked).

### Statistical Analysis

Data were analyzed using SPSS (version 20.0). Continuous variables and categorical variables were summarized as the geometric mean ± standard deviation and proportions, respectively. Correlations between health conditions, lifestyle factors and depression were assessed by spearman correlation coefficient. Differences in health conditions, lifestyle factors between the depressive and non-depressive participants were examined by χ2 tests for categorical variables. Logistic regression was to examine the effect of health conditions and lifestyle factors on depression, and risk factors were evaluated in terms of the odds ratios (ORs) and 95% confidence intervals (CIs). The statistical significance was at *P* < 0.05.

## Results

Table 1 presented the characteristics of participants included in the analysis. Of 3,300 participants, 1,327 were males (40.21%) and 1,973 were females (59.79%). The mean age of participants was 50.63 ± 10.38. A large proportion of participants had a senior high school or higher education (38.34%), lived in the rural district (56.15%), and had an occupation (69.37%). The majority of participants (95.86%) were married or cohabiting with their spouses. Of 3,300 participants, 1,362 individuals (42.58%) earned <599 CNY per month, 1,494 individuals (46.70%) earned 600–1,999 RMB per month, and 343 individuals (10.72%) earned more than 2,000 RMB per month.

**Table 1:** Socio-economic and demographic characteristics of analysis subjects.

	**Total** **(*****N*** **=** **3,300)**		**Men** **(*****n*** **=** **1,327)**		**Women** **(*****n*** **=** **1,973)**	
	***N*/mean**	**%/SD**	***N*/mean**	**%/SD**	***N*/mean**	**%/SD**
Age	50.63	10.38	50.41	10.62	50.78	10.22
Area						
Urban	14,47	43.85	537	40.47	910	46.12
Rural	1,853	56.15	790	59.53	1,063	53.88
Education						
Illiterate/elementary school	930	28.30	251	18.99	679	34.57
Junior high school	1,096	33.35	466	35.25	630	32.08
Senior high school or higher	1,260	38.34	605	45.76	655	33.35
Marriage						
Unmarried	135	4.14	32	2.44	103	5.29
Married	3,123	95.86	1,279	97.56	1,844	94.71
Income						
≤ 599	1,362	42.58	442	34.1	920	48.34
600–1,999	1,494	46.70	611	47.15	883	46.4
≥2,000	343	10.72	243	18.75	100	5.25
Occupation						
No occupation	987	30.63	337	25.98	650	33.77
Any kind occupation	2235	69.37	960	74.02	1,275	66.23

Table 2 showed correlations between health conditions, lifestyle factors, and depression. Diabetic status was independently and positively associated with Zung scores (*P* < 0.001), smoking and drinking were independently and negatively associated with Zung scores (*P* < 0.001).

**Table 2:** Spearman correlation between health conditions, lifestyle factors, and depression.

	**Zung scores**	**Diabetic status**	**BMI**	**Physical activity**	**Smoking**	**Drinking**
Zung scores	1.000	0.106^***^	−0.009	0.016	−0.108^***^	−0.080^***^
Diabetic status	0.106^***^	1.000	0.168^***^	0.416^***^	−0.051^**^	−0.066^**^
BMI	−0.009	0.168^**^	1.000	0.036^*^	−0.053^**^	−0.002
Physical activity	0.016	0.416^***^	0.036^*^	1.000	−0.025	−0.048^**^
Smoking	−0.108^***^	−0.051^**^	−0.053^**^	−0.025	1.000	0.471^***^
Drinking	−0.080^***^	−0.066^***^	−0.002	−0.048^**^	0.471^***^	1.000

*The data reported in the table are correlation coefficients*.

* *P < 0.05*;

** *P < 0.01*,

*** *P < 0.001*.

Table 3 showed health variables, health condition variables and depression variables for the participants by gender. The mean Zung scores for all participants, male participants, and female participants were 29.73 ± 7.57, 28.89 ± 7.30, 30.30 ± 7.70, respectively. For all participants, the prevalence of depression was significantly different between those who was diagnosed normoglycaemia (*n* = 71, 4.25%), pre-diabetes (*n* = 55, 7.13%), newly diagnosed diabetes (*n* = 20, 5.17%), and previously diagnosed diabetes (*n* = 33, 7.02%). In addition, the prevalence of depression was significantly different between underweight participants (*n* = 81, 5.97%), normal-weight participants (*n* = 3, 16.67%), and overweight/obese participants (*n* = 95, 4.93%). For men, the prevalence of depression was significantly different between those who was diagnosed normoglycaemia (*n* = 19, 2.77%), pre-diabetes (*n* = 20, 6.67%), newly diagnosed diabetes (*n* = 7, 4.02%), and previously diagnosed diabetes (*n* = 11, 6.63%).

**Table 3:** The characteristics of health conditions, lifestyle factors, and depression in analysis subjects.

	**Total**			**Men**			**Women**		
	***N* (%)**	**Depression** **(%)**	***P*-value**	***N* (%)**	**Depression (%)**	***P*-value**	***N* (%)**	**Depression (%)**	***P*-value**
**Health conditions**									
Diabetic status			<0.05			<0.05			0.36
Normoglycaemia	1,672 (50.67)	71 (4.25)		687 (51.77)	19 (2.77)		985 (49.92)	52 (5.28)	
Pre-diabetes	771 (23.36)	55 (7.13)		300 (22.61)	20 (6.67)		471 (23.87)	35 (7.43)	
Newly diagnosed diabetes	387 (11.73)	20 (5.17)		174 (13.11)	7 (4.02)		213 (10.8)	13 (6.10)	
Previously diagnosed diabetes	470 (14.24)	33 (7.02)		166 (12.51)	11 (6.63)		304 (15.41)	22 (7.24)	
BMI			<0.05			0.33			0.10
Normal weight	1356 (41.09)	81 (5.97)		543 (40.92)	27 (4.97)		813 (41.21)	54 (6.64)	
Underweight	18 (0.55)	3 (16.67)		9 (0.68)	1 (11.11)		9 (0.46)	2 (22.22)	
Overweight	1926 (58.36)	95 (4.93)		775 (58.4)	29 (3.74)		1151 (58.34)	66 (5.73)	
**Lifestyle factors**									
Physical activity			0.35			0.19			0.84
No	3036 (92)	168 (5.53)		1218 (91.79)	55 (4.52)		1818 (92.14)	113 (6.22)	
Yes	264 (8)	11 (4.17)		109 (8.21)	2 (1.83%)		155 (7.86)	9 (5.81)	
Smoking			0.80			0.28			0.23
No	2611 (79.12)	143 (5.48)		675 (50.87)	25 (3.70)		1936 (98.12)	118 (6.10)	
Yes	689 (20.88)	36 (5.22)		652 (49.13)	32 (4.91)		37 (1.88)	4 (10.81)	
Drinking			0.25			0.98			0.48
No	2636 (79.88)	149 (5.65)		696 (52.45)	30 (4.31)		1940 (98.33)	119 (6.13)	
Yes	664 (20.12)	30 (4.52)		631 (47.55)	27 (4.28)		33 (1.67)	3 (9.09)	

Logistic regression analysis was adopted to explore the association of health conditions and lifestyle factors on depression, shown in Table 4. Model 3 indicated that diabetic status, physical activity, and area were significant risk factors for depression. Specifically, pre-diabetes participants had about 1.53 times greater risk for depression compared to participants with normoglycaemia (95% CI, 1.04–2.27), participants with regular physical activity more less likely become depressed than participants with irregular physical activity: the OR was 0.39 (95% CI, 0.17–0.89), the OR for depression of rural districts compared with urban districts was 0.36 (95% CI, 0.24–0.54).

**Table 4:** Odds ratio (OR) and 95% CI of health conditions, lifestyle factors for depression in all subjects in logistic regression models.

	**Model 1**	**Model 2**	**Model 3**
Diabetic status			
Normoglycaemia	1.00	1.00	1.00
Pre-diabetes	1.85 (1.28–2.67)^**^	1.84 (1.28–2.66)^**^	1.53 (1.04–2.27)^*^
Newly diagnosed diabetes	1.32 (0.79–2.21)	1.33 (0.80–2.23)	1.16 (0.67–2)
Previously diagnosed diabetes	1.80 (1.17–2.76)^**^	2.43 (1.49–3.99)^***^	1.82 (1.00–3.30)
BMI			
Normal weight	1.00	1.00	1.00
Underweight	3.39 (0.95–12.04)	3.14 (0.87–11.33)	2.73 (0.59–12.74)
Overweight	0.75 (0.55–1.02)	0.75 (0.55–1.02)	0.81 (0.58–1.13)
Physical activity			
No		1.00	1.00
Yes		0.44 (0.21–0.92)^**^	0.39 (0.17–0.89)^*^
Smoking			
No		1.00	1.00
Yes		1.05 (0.68–1.6)	1.11 (0.7–1.76)
Drinking			
No		1.00	1.00
Yes		0.82 (0.52–1.29)	0.91 (0.55–1.49)
Area			
Urban			1.00
Rural			0.36 (0.24–0.54)^**^
Age			1.00 (0.98–1.02)
Education			
Illiterate/elementary school			1.00
Junior high school			0.76 (0.47–1.23)
Senior high school or higher			0.61 (0.36–1.04)
Marriage			
Unmarried			1.00
Married			1.02 (0.49–2.09)
Income			
≤599			1.00
600–1,999			0.95 (0.62–1.45)
≥2,000			0.86 (0.44–1.7)
Occupation			
No occupation			1.00
Any kind occupation			1.00 (0.64–1.56)

*The data reported in the table are OR with 95% CIs in parentheses*.

* *P < 0.05*;

** *P < 0.01*,

*** *P < 0.001*.

Table 5 presented risk factors on depression by gender. For men, diabetic status, physical activity, and area were significant risk factors for the depression. Specifically, participants with pre-diabetes and previously diagnosed diabetes had about 2.49 times (95% CI, 1.25–4.97) and 4.44 times (95% CI, 1.58–12.48) greater risk for depression compared to participants with normoglycaemia, the OR of having depression for those with regular activity compared to those with irregular activity was 0.07 (95% CI, 0.01–0.61), the OR of having depression of rural compared with urban was 0.20 (95% CI, 0.10–0.42). For women, BMI and area were a statistically significant association to the depression. Specifically, underweight participants had had about 5.7 times (95% CI, 1.04–30.71) greater risk for depression compared to participants with normal weight, the OR of having depression of rural districts compared with urban districts was 0.52 (95% CI, 0.31–0.86).

**Table 5:** Odds ratio (OR) and 95% CI of health conditions, lifestyle factors for depression in men and women in logistic regression models.

	**Men**	**Women**
Diabetic status		
Normoglycaemia	1.00	1.00
Pre-diabetes	2.49 (1.25–4.97)^**^	1.15 (0.7–1.88)
Newly diagnosed diabetes	1.37 (0.51–3.66)	1.04 (0.53–2.04)
Previously diagnosed diabetes	4.44 (1.58–12.48)^**^	1.06(0.5–2.24)
BMI		
Normal weight	1.00	1.00
Underweight	-	5.66 (1.04–30.71)^*^
Overweight	0.66 (0.37–1.2)	0.83 (0.55–1.25)
Physical activity		
No	1.00	1.00
Yes	0.07 (0.01–0.61)^*^	0.73 (0.29–1.88)
Smoking		
No	1.00	1.00
Yes	1.64 (0.89–3.03)	1.27 (0.36–4.49)
Drinking		
No	1.00	1.00
Yes	1.05 (0.56–1.96)	1.84 (0.52–6.5)
Area		
Urban	1.00	1.00
Rural	0.2 (0.1–0.42)^***^	0.52 (0.31–0.86)^**^
Age	0.99 (0.96–1.02)	1.02 (0.99–1.04)
Education		
Illiterate/elementary school	1.00	1.00
Junior high school	1.68 (0.52–5.45)	0.79 (0.45–1.4)
Senior high school or higher	0.93 (0.26–3.3)	0.72 (0.37–1.37)
Marriage		
Unmarried	1.00	1.00
Married	1.19 (0.15–9.62)	0.99 (0.45–2.19)
Income		
≤599	1.00	1.00
600–1,999	1.15 (0.51–2.63)	0.96 (0.57–1.6)
≥2,000	1.47 (0.52–4.13)	0.32 (0.07–1.44)
Occupation		
No occupation	1.00	1.00
Any kind occupation	1.32 (0.53–3.29)	0.92 (0.55–1.55)

*The data reported in the table are OR with 95% CIs in parentheses*.

* *P < 0.05*;

** *P < 0.01*,

*** *P < 0.001*.

## Discussion

The present study analyzed how the health conditions and lifestyle factors influenced depression among adults in Qingdao, China. The results showed that health conditions and lifestyle factors were the important risk factors of depression.

### Health Conditions

The previous studies mainly investigated the association between diabetes and depression, and results showed that the prevalence of depression was significantly higher risk in subjects with diabetes compared with the non-diabetic population (16–19). Our study further analyzed the relationship between different stages of diabetes and depression, and results were not completely consistent with other studies (5, 5, 20, 21). Our finding showed that pre-diabetes, but not newly diagnosed diabetes and previously diagnosed diabetes, was significantly associated with depression. There were several possible explanations for elevated depressive symptoms in the pre-diabetes population in this study. One explanation was that the “psychological burden hypothesis,” which indicated that the burden of knowing diabetes and concomitant manage, could cause some negative effects on psychological feelings (22). Another explanation was that the population were mainly composed of middle-aged participants in our study, they might suffer from other diseases, thus leading them to have depressive symptoms even in the pre-diabetes stage. Meanwhile, we had to pay attention to a phenomenon that the detection rate of pre-diabetes in China was as high as 50.1% in 2013 (23), and most people had no enough knowledge of this abnormal state so that they were more likely to be in anxiety or even depression state.

Our study found that gender difference of association of diabetes with the development of depression. Participants with pre-diabetes and previously diagnosed diabetes had a greater risk in men, whereas, diabetic status was not associated with depression in women. Some early studies have reported that women generally have higher prevalence of depression compared to men (24, 25), and this has been attributed to hormone changes (e.g., testosterone, estrogen) (26), the experience of more interpersonal stressors (27) and greater tendency to ruminate (28). According to the results of descriptive statistics, our study was consistent with previous studies, where women had higher levels of depression at all stages of diabetic status compared to men. Therefore, diabetic status was not a risk factor for depression in women, because women had the higher prevalence to be associated with numerous biological, social, and psychologic factors, regardless of the presence of diabetes.

This study was not clear as to whether BMI was a risk factor for depression in the general population. However, several earlier studies consistently reported that BMI and depression were associated, yet the relationship was controversial (29–32). Besides, previous studies also shown that depression with BMI category also was detected differences between men and women, higher BMI in women and lower BMI in men resulted in more serious depression (33). Our results differed from those of several other studies that had reported, which underweight in women was a critical factor for developing depression (95%CI, 1.04–30.71). There were three possible explanations for this result. One explanation was that underweight in the 40–59 year age group than other age groups were more likely to become depressed (34), and the average age of this study was 50.63 ± 10.38. Another explanation was that the underweight subgroup could include those who ate disorder or have to be in the stage of losing weight [Jorm et al. (35)]. Furthermore, according to with “Jolly Fat” hypothesis, overweight had a lower risk for depression due to several possible mechanisms that might include higher consumption of certain nutrients that helped reduce or prevent depressive symptoms (36, 37).

### Lifestyle Factors

In this study, the lack of physical activity was a risk factor for depression. Similarly, several previous cohort studies showed that physical activity could confer protection against the emergence of depression (38–40). At a clinical level, a study proved that 63% of exercise participants had lower severity of depression than the control group and approximately 20% reduction in the severity of depressive (41). However, our study also certified the significance might vary according to gender, physical activity showed protective effects against depressive symptoms in men, but not in women. A study also predicted that leisure-time exercise and walking or cycling during commuting to work were associated with better mental health in men (42).

Because nicotine could damage certain pathways in the brain that regulate mood, many prior studies had examined that smokers were more likely to develop incident depression vs. non-smokers (43–47) and gender differences existed in their associations (48–50). In contrast, our findings were inconsistent with other studies; we did not observe a similar association in smoking and depression after controlling confounding variables. The reason could be explained that previous findings linking smoking to higher levels of depression might be due to residual confounding, a shared vulnerability to both depression and smoking behavior, or reverse causality (51).

Our findings contradicted a large body of other studies that had documented drinking could lead to depression given the various psychological effects of alcohol and impacted on mental health (52–54). Results in our study were consistent with the UK based study (55) and the Whitehall II cohort of British civil servants (56), concluded that there was no association between drinking and risk of depression. A partial explanation for the discrepant results between the prior studies, ours might be that moderate doses of alcohol did not cause many problems and was effective in reducing stress (57). Especially in countries like China, drinking had always been common, and most people felt comfortable with drinking being part of the Chinese liquor culture.

This study suffered from a few limitations. Firstly, this study was a cross-sectional study that did not reflect the underlying mechanisms between depression and other factors. Therefore, a follow-up study was necessary. Second, the analysis of depression symptoms was by ZSDS only. It would be more reliable if this study analyzes depression combined with other scales. Thirdly, the data of our study was collected from Qingdao Diabetes Prevention Program, and some variables like physical activity, smoking and drinking status had not been designed in detail in the questionnaire.

## Conclusion

This study found that diabetic status and physical activity were significantly associated with the development of depression. Meanwhile, the statistical differences of gender on depression were found that men with prediabetes or diabetes had a high risk for depression, underweight in women was a risk factor for depression, and physical activity had protective effects against depression in men. Thus, in future studies, researchers should pay more attention to persons who comply with these factors mentioned above, and intervene in those factors that play a significant role in the prevention of depression.

## Data Availability Statement

The datasets generated for this study are available on request to the corresponding author.

## Ethics Statement

The studies involving human participants were reviewed and approved by this study was approved by the Ethics Committee and Institutional Review Board of Qingdao Centers for Disease Control and Prevention. The authors declare that all procedures contributing to this work comply with the ethical standards of the relevant national and institutional committees on human experimentation and with the Helsinki Declaration of 1975, as revised in 2008. The patients/participants provided their written informed consent to participate in this study.

## Author Contributions

JS and ZB designed this analysis. NC and ZB analyzed the data and drafted the study. JC and XX drafted tables. NC, BA, JC, XX, DL, LB, MZ, and DW revised the manuscript critically for intellectual content. All authors gave intellectual input to the study and approved the final version of the manuscript.

## Conflict of Interest

The authors declare that the research was conducted in the absence of any commercial or financial relationships that could be construed as a potential conflict of interest.

## References

[1] World Health Organization. The Global Burden of Disease: 2004 update. World Health Organization (2008).

[2] Charlson FJ Baxter AJ Cheng HG Shidhaye R Whiteford HA. The burden of mental, neurological, and substance use disorders in China and India: a systematic analysis of community representative epidemiological studies. Lancet. (2016) 388:376–89. 10.1016/S0140-6736(16)30590-6 27209143

[3] Wiltink J Michal M Wild PS Zwiener I Blettner M Münzel T. Associations between depression and different measures of obesity (BMI, WC, WHtR, WHR). BMC Psychiatry. (2013) 13:223. 10.1186/1471-244X-13-223 24028572 PMC3849983

[4] Bisschop MI Kriegsman DM Deeg DJ Beekman AT Van TW. The longitudinal relation between chronic diseases and depression in older persons in the community: the Longitudinal Aging Study Amsterdam. J Clin Epidemiol. (2004) 57:187–94. 10.1016/j.jclinepi.2003.01.001 15125629

[5] Nouwen A Nefs G Caramlau I Connock M Winkley K Lloyd CE. Prevalence of depression in individuals with impaired glucose metabolism or undiagnosed diabetes. Diabetes Care. (2011) 34:752. 10.2337/dc10-1414 21357362 PMC3041222

[6] Dolatian A Arzaghi SM Qorbani M Pishva H. The relationship between body mass index (BMI) and depression according to the rs16139NPY Gene. Iran J Psychiatry. (2017) 12:201–5 29062372 PMC5640582

[7] Lopresti AL Hood SD Drummond PD. A review of lifestyle factors that contribute to important pathways associated with major depression: diet, sleep, and exercise. J Affect Disord. (2013) 148:12–27. 10.1016/j.jad.2013.01.014 23415826

[8] Lo A. Lifestyle factors on depression. In: Pachana N, editor. Encyclopedia of Geropsychology. Springer (2015) 10.1007/978-981-287-080-3_279-1

[9] Behmani RK Upmanyu M. Effect of gender and age on depression among adolescents. Acta Orthop Belg. (2015) 76:144–9.

[10] Saygin GD Huseyin KH Esenkaya Ö Emin OM Halac G Asil T. Influences of socio-demographics on depression and anxiety in patients with complex partial and tonic-clonic seizures. Med Glas. (2014) 11:356–60. 25082253

[11] Tanaka H Sasazawa Y Suzuki S Nakazawa M Koyama H. Health status and lifestyle factors as predictors of depression in middle-aged and elderly Japanese adults: a seven-year follow-up of the Komo-Ise cohort study. BMC Psychiatry. (2011) 11:20. 10.1186/1471-244X-11-20 21294921 PMC3041738

[12] Zung WWK Richards CB Short MJ. Self-rating depression scale in an outpatient clinic: further validation of the SDS. Arch Gen Psychiatry. (1965) 13:508–15. 10.1001/archpsyc.1965.01730060026004 4378854

[13] Biggs JT Wylie LT Ziegler VE. Validity of the Zung Self-rating depression scale. Br J Psychiatry. (1978) 132:381–5. 10.1192/bjp.132.4.381 638392

[14] Fountoulakis KN Lacovides A Samolis S Kleanthous S Kaprinis SG Kaprinis GS. Reliability, validity and psychometric properties of the Greek translation of the zung depression rating scale. BMC Psychiatry. (2001) 1:6. 10.1186/1471-244X-1-6 11806757 PMC64635

[15] World Health Organization. Definition and Diagnosis of Diabetes Mellitus and Intermediate Hyperglycaemia: Report of a WHO/IDF Consultation. Geneva: World Health Organization (2006).

[16] Nichols GA Brown JB. Unadjusted and adjusted prevalence of diagnosed depression in type 2 diabetes. Diabetes Care. (2003) 26:744. 10.2337/diacare.26.3.744 12610032

[17] Anderson R Freedland K Clouse R Lustman P. The prevalence of comorbid depression in adults with diabetes: a meta-analysis. Diabetes Care. (2001) 24:1069. 10.2337/diacare.24.6.1069 11375373

[18] Ali S Stone MA Peters JL Davies MJ Khunti K. The prevalence of co-morbid depression in adults with Type 2 diabetes: a systematic review and meta-analysis. Diabet Med. (2010) 23:1165–73. 10.1111/j.1464-5491.2006.01943.x 17054590

[19] Khamseh ME Baradaran HR Rajabali H. Depression and diabetes in Iranian patients: a comparative study. Int J Psychiat Med. (2007) 37:81. 10.2190/FP64-82V3-1741-842V 17645200

[20] Knol MJ Heerdink ER Egberts ACG Geerlings MI Gorter KJ Numans ME. Depressive symptoms in subjects with diagnosed and undiagnosed type 2 diabetes. Psychosom Med. (2007) 69:300. 10.1097/PSY.0b013e31805f48b9 17470664

[21] Sun JC Xu M Lu JL Bi YF Mu YM Zhao JJ. Associations of depression with impaired glucose regulation, newly diagnosed diabetes and previously diagnosed diabetes in Chinese adults. Diabetic Med. (2015) 32:935–43. 10.1111/dme.12649 25439630

[22] Talbot F Nouwen A. A review of the relationship between depression and diabetes in adults: is there a link? Diabetes Care. (2000) 23:1556–62. 10.2337/diacare.23.10.1556 11023152

[23] Yu X Limin W Jiang H Yufang B Mian L Tiange W. Prevalence and control of diabetes in Chinese adults. JAMA. (2013) 310:948–59. 10.1001/jama.2013.168118 24002281

[24] Amenson CS Lewinsohn PM. An investigation into the observed sex difference in prevalence of unipolar depression. J Abnorm Psychol. (1981) 90:1–13. 10.1037/0021-843X.90.1.1 6973575

[25] Lewinsohn PM Pettit JW Joiner TJ Seeley JR. The symptomatic expression of major depressive disorder in adolescents and young adults. J Abnorm Psychol. (2003) 112:244–52. 10.1037/0021-843X.112.2.244 12784834

[26] de Jonge P Roy JF Saz P Marcos G Lobo A. Prevalent and incident depression in community-dwelling elderly persons with diabetes mellitus: results from the ZARADEMP project. Diabetologia. (2006) 49:2627–33. 10.1007/s00125-006-0442-x 17019601

[27] Shih JH Eberhart NK Hammen CL Brennan PA. Differential exposure and reactivity to interpersonal stress predict sex differences in adolescent depression. J Clin Child Adolesc Psychol. (2006) 35:103–15. 10.1207/s15374424jccp3501_9 16390306

[28] Nolen-Hoeksema S. The role of rumination in depressive disorders and mixed anxiety/depressive symptoms. J Abnorm Psychol. (2000) 109:504–11. 10.1037/0021-843X.109.3.504 11016119

[29] Jinseok K Jin-Won N Jumin P Young Dae K. Body mass index and depressive symptoms in older adults: a cross-lagged panel analysis. PLoS ONE. (2014) 9:e114891. 10.1371/journal.pone.0114891 25501372 PMC4263712

[30] Carter JD Assari S. Sustained obesity and depressive symptoms over 6 years: race by gender differences in the health and retirement study. Front Aging Neurosci. (2016) 8:312. 10.3389/fnagi.2016.00312 28101050 PMC5209386

[31] Dong C Sanchez LE Price RA. Relationship of obesity to depression: a family-based study. Int J Obesity. (2004) 28:790–5. 10.1038/sj.ijo.0802626 15024401

[32] Noh JW Kwon YD Park J Kim J. Body mass index and depressive symptoms in middle aged and older adults. BMC Public Health. (2015) 15:1–7. 10.1186/s12889-015-1663-z 25884564 PMC4383216

[33] Carpenter K Hasin DD Faith M. Relationships between obesity and DSM-IV major depressive disorder, suicide ideation, and suicide attempts: results from a general population study. Am J Public Health. (2000) 90:251–7. 10.2105/AJPH.90.2.251 10667187 PMC1446144

[34] Chen Y Jiang Y Mao Y. Association between obesity and depression in Canadians. J Womens Health. (2009) 18:1687. 10.1089/jwh.2008.1175 19785572

[35] Jorm AF Korten AE Christensen H Jacomb PA Rodgers B Parslow RA. Association of obesity with anxiety, depression and emotional well-being: a community survey. Aust N Z J Public Health. (2010) 27:434–40. 10.1111/j.1467-842X.2003.tb00423.x 14705308

[36] Yim G Ahn Y Cho J Chang Y Ryu S Lim JY. The “Jolly Fat” effect in middle-aged Korean women. J Womens Health. (2017) 26:1236–43. 10.1089/jwh.2016.6254 28922089

[37] Crisp AH Mcguiness B. Jolly fat: relation between obesity and psychoneurosis in general population. Br Med J. (1976) 1:7–9. 10.1136/bmj.1.6000.7 1247732 PMC1638245

[38] Harris AHS Cronkite R Moos R. Physical activity, exercise coping, and depression in a 10-year cohort study of depressed patients. J Affect Disord. (2006) 93:79–85. 10.1016/j.jad.2006.02.013 16545873

[39] Schuch FB Vancampfort D Firth J Rosenbaum S Ward PB Silva ES. Physical activity and incident depression: a meta-analysis of prospective cohort studies. Am J Psychiatry. (2018) 175:2011–8. 10.1176/appi.ajp.2018.17111194 29690792

[40] Uemura K Makizako H Lee S Doi T Lee S Tsutsumimoto K. Behavioral protective factors of increased depressive symptoms in community-dwelling older adults: a prospective cohort study. Int J Geriatr Psychiatry. (2018) 33:e234–41. 10.1002/gps.4776 28841238

[41] Bridle C Spanjers K Patel S Atherton NM Lamb SE. Effect of exercise on depression severity in older people: systematic review and meta-analysis of randomised controlled trials. Br J Psychiatry. (2012) 201:180–5. 10.1192/bjp.bp.111.095174 22945926

[42] Ohta M Mizoue T Mishima N Ikeda M. Effect of the physical activities in leisure time and commuting to work on mental health. J Occup Health. (2007) 49:46–52. 10.1539/joh.49.46 17314466

[43] Tully EC Iacono WG Matt MG. Changes in genetic and environmental influences on the development of nicotine dependence and major depressive disorder from middle adolescence to early adulthood. Dev Psychopathol. (2010) 22:831. 10.1017/S0954579410000490 20883585 PMC2951615

[44] Lam TH Li ZB Ho SY Chan WM Ho KS Li MP. Smoking and depressive symptoms in Chinese elderly in Hong Kong. Acta Psychiatr Scand. (2010) 110:195–200. 10.1111/j.1600-0447.2004.00342.x 15283739

[45] Dierker LC Avenevoli S Merikangas KR Flaherty BP Stolar M. Association between psychiatric disorders and the progression of tobacco use behaviors. J Am Acad Child Adolesc Psychiatry. (2001) 40:1159–67. 10.1097/00004583-200110000-00009 11589528

[46] Phillip B. Smoking cessation improves anxiety depression. Practitioner. (2014) 258:5. 24791405

[47] Boden JM Fergusson DM John LH. Cigarette smoking and depression: tests of causal linkages using a longitudinal birth cohort. Br J Psychiatry. (2010) 196:440–6. 10.1192/bjp.bp.109.065912 20513853

[48] Suzuki H Kadota A Okuda N Hayakawa T Nishi N Nakamura Y. Socioeconomic and lifestyle factors associated with depressive tendencies in general Japanese men and women: NIPPON DATA2010. Environ Health Prev. (2019) 24:37. 10.1186/s12199-019-0788-6 31138144 PMC6540356

[49] Tsoh JY Lam JN Delucchi KL Hall SM. Smoking and depression in Chinese Americans. Am J Med Sci. (2003) 326:187–91. 10.1097/00000441-200310000-00007 14557732

[50] Khaled SM Andrew B Exner DV Patten SB. Cigarette smoking, stages of change, and major depression in the Canadian population. Can J Psychiatry. (2009) 54:204–8. 10.1177/070674370905400309 19321025

[51] Kendler KS Neale MC Maclean CJ Heath AC Eaves LJ Kessler RC. Smoking and major depression. A causal analysis. Arch Gen Psychiatry. (1993) 50:36–43. 10.1001/archpsyc.1993.01820130038007 8422220

[52] Huang R Ho SY Wang MP Lo WS Lam TH. Reported alcohol drinking and mental health problems in Hong Kong Chinese adolescents. Drug Alcohol Depend. (2016) 164:47–54. 10.1016/j.drugalcdep.2016.04.028 27177803

[53] Adams RE Boscarino JA Sandro G. Alcohol use, mental health status and psychological well-being 2 years after the World Trade Center attacks in New York City. Am J Drug Alcohol Abuse. (2006) 32:203–24. 10.1080/00952990500479522 16595324 PMC2746081

[54] Lange S Quere M Shield K Rehm J Popova S. Alcohol use and self-perceived mental health status among pregnant and breastfeeding women in Canada: a secondary data analysis. BJOG. (2016) 123:900–9. 10.1111/1471-0528.13525 26344418

[55] Haynes JC Farrell M Singleton N Meltzer H Araya R Lewis G. Alcohol consumption as a risk factor for anxiety and depression: results from the longitudinal follow-up of the National Psychiatric Morbidity Survey. Br J Psychiatry. (2005) 187:544–51. 10.1192/bjp.187.6.544 16319407

[56] Bell S Britton A. Drinking pattern during midlife and risk of developing depression during 28 years of follow-up: a prospective cohort study. Drug Alcohol Depend. (2015) 155:111–7. 10.1016/j.drugalcdep.2015.08.008 26321670

[57] García-Esquinas E Ortolá R Galán I Soler-Vila H Laclaustra M Rodríguez-Artalejo F. Moderate alcohol drinking is not associated with risk of depression in older adults. Sci Rep. (2018) 8:11512. 10.1038/s41598-018-29985-4 30065286 PMC6068095

